# Diagnostic Accuracy of Liquid Biomarkers in Airway Diseases: Toward Point-of-Care Applications

**DOI:** 10.3389/fmed.2022.855250

**Published:** 2022-06-06

**Authors:** Vivianne Landry, Patrick Coburn, Karen Kost, Xinyu Liu, Nicole Y. K. Li-Jessen

**Affiliations:** ^1^Faculty of Medicine, University of Montreal, Montreal, QC, Canada; ^2^School of Communication Sciences & Disorders, McGill University, Montreal, QC, Canada; ^3^Department of Otolaryngology-Head & Neck Surgery, McGill University, Montreal, QC, Canada; ^4^Department of Mechanical & Industrial Engineering, University of Toronto, Toronto, ON, Canada; ^5^Department of Biomedical Engineering, McGill University, Montreal, QC, Canada; ^6^The Research Institute of the McGill University Health Centre, Montreal, QC, Canada

**Keywords:** biomarkers, airway diseases, point-of-care, diagnostic accuracy, COVID-19

## Abstract

**Background:**

Liquid biomarkers have shown increasing utility in the clinical management of airway diseases. Salivary and blood samples are particularly amenable to point-of-care (POC) testing due to simple specimen collection and processing. However, very few POC tests have successfully progressed to clinical application due to the uncertainty and unpredictability surrounding their diagnostic accuracy.

**Objective:**

To review liquid biomarkers of airway diseases with well-established diagnostic accuracies and discuss their prospects for future POC applications.

**Methodology:**

A literature review of publications indexed in Medline or Embase was performed to evaluate the diagnostic accuracy of liquid biomarkers for chronic obstructive pulmonary disease (COPD), asthma, laryngopharyngeal reflux (LPR), and COVID-19.

**Results:**

Of 3,628 studies, 71 fulfilled the inclusion criteria. Sputum and blood eosinophils were the most frequently investigated biomarkers for the management of asthma and COPD. Salivary pepsin was the only biomarker with a well-documented accuracy for the diagnosis of LPR. Inflammatory blood biomarkers (e.g., CRP, D-dimers, ferritin) were found to be useful to predict the severity, complications, and mortality related to COVID-19 infection.

**Conclusion:**

Multiple liquid biomarkers have well-established diagnostic accuracies and are thus amenable to POC testing in clinical settings.

## Introduction

**FIGURE 1:**
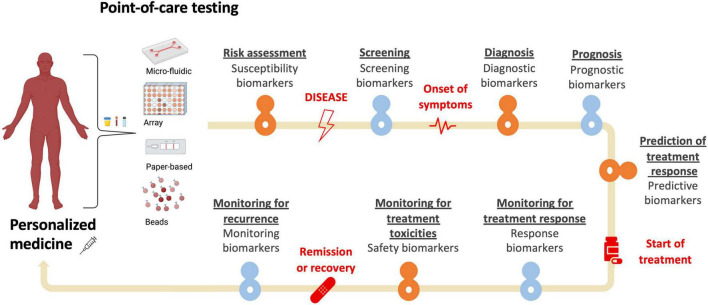
Types of biomarkers.

Rapid advancements in genomics, transcriptomics, proteomics, metabolomics, and other “-omics” technologies have allowed for the identification of a vast array of new biomarkers that can be used as tools for improving prevention, diagnosis, prognosis, and management of both non-communicable and communicable airway diseases. It is postulated that this “biomarker revolution” has been paving the way for precision medicine (1). The FDA-NIH Biomarker Working Group defines biomarkers as “*defined characteristics that [are] measured as indicator[s] of normal biological processes, pathogenic processes or responses to an exposure or intervention, including therapeutic interventions*.” (2) Biomarkers can be useful at all stages of disease progression (Figure 1), including:

(1) For risk assessment of diseases (susceptibility biomarkers).
(2) For detection of diseases (screening and diagnostic biomarkers).
(3) For establishing prognosis (prognostic biomarkers).
(4) For planning treatment (predictive biomarkers).
(5) For monitoring therapeutic and adverse effects related to treatment (response or safety biomarkers).
(6) For assessment of disease status or detection of disease recurrence (monitoring biomarkers) (2).

Point-of-care (POC) technology provides a convenient alternative to centralized laboratory analysis of biological samples. POC technology allows certain molecular and antigen tests to be performed and analyzed rapidly anywhere in a near-patient setting, such as in patients’ homes or at their bedsides (3). Biofluids or liquid biomarkers are particularly amenable to POC testing. Not only are biofluids a rich source of molecular proteins and peptides, but their collection is also relatively simple and minimally invasive. For instance, salivary biomarkers have been proposed to detect and monitor inflammatory and infectious diseases including chronic inflammatory disorders (e.g., inflammatory bowel and periodontal conditions) and sexually transmitted infections (e.g., HIV and HPV) (4–7). The diagnostic accuracy of molecular biomarkers, however, need to be rigorously established before they transition to the convenience of POC testing.

**FIGURE 2:**
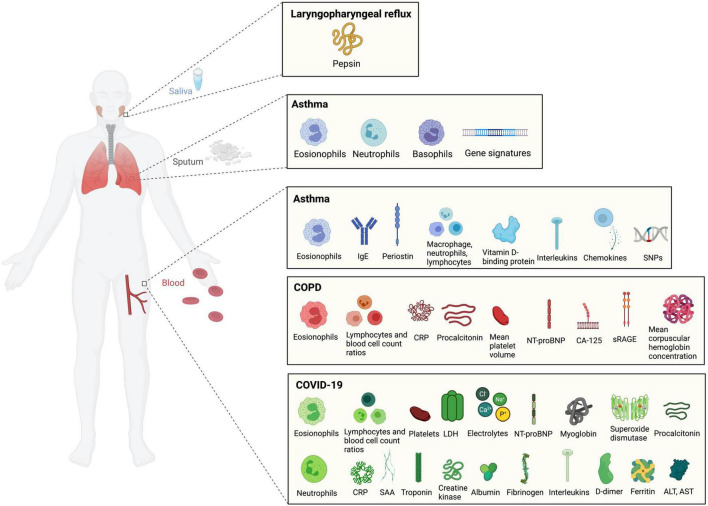
Liquid biomarkers of airway diseases.

A growing number of liquid biomarkers have been associated with airway diseases. The airway tract is comprised of the organs involved in respiration, speech, and deglutition, including the nose, oral cavity, pharynx, larynx, trachea, bronchi, and lungs (8). Airway and pulmonary diseases may include obstructive airway conditions such as asthma or chronic obstructive pulmonary disease (COPD), infectious diseases such as COVID-19, and chronic chemical irritation such as airway reflux, also known as laryngopharyngeal reflux (LPR). The human airway is lined with airway surface liquid, which plays an important role in the clearing of environmental toxins and defense against foreign particles (9). Local biofluids (e.g., saliva, sputum, mucus, etc.) thus naturally lend themselves as a convenient source of airway liquid biomarkers (10–13). Peripheral biofluids (e.g., blood, serum, urine, etc.) may also provide valuable information regarding systemic processes that contribute to airway diseases (14–16) (Figure 2).

Although biofluid samples can be easily obtained in physicians’ offices or outside of the healthcare setting (e.g., through sputum induction), POC testing has yet to be made widely available for the management of airway diseases. A long-standing and legitimate barrier to the implementation of POC testing is the uncertainty surrounding the diagnostic accuracy of liquid biomarker measurements (17). In this review, we aim to provide peer-reviewed evidence regarding the clinical utility of airway liquid biomarkers with well-established diagnostic accuracies that would be readily translated to POC testing. We first survey recent literature on liquid biomarkers in COPD, asthma, airway reflux, and COVID-19 and their diagnostic accuracies. Then, we discuss several emerging POC platforms under development and present prospects of future POC applications for liquid biomarkers in airway diseases.

## Methods

### Selection of Airway Conditions

Four common airway and pulmonary conditions, namely asthma, COPD, LPR, and COVID-19, were included in this review. The biomarkers of these four conditions were the most abundantly researched and could be tested through biomarker detection devices available on the market.

#### Search Strategy

A review of the literature was performed to identify relevant studies reporting on the diagnostic accuracy of liquid biomarkers for airway diseases. The PRISMA (Preferred Reporting Items for Systematic reviews and Meta-analyses) framework was used to guide the search and the reporting of the review (18). Two databases, namely Medline and Embase, were searched for references published from 2000 to 2021, respectively. The following terms were searched: *“biological marker”; “biomarker”; “biologic marker”; “diagnostic accuracy”; “sensitivity and specificity”; “sensitivity”; “specificity”; “laryngopharyngeal reflux”; “proximal reflux”; “hypopharyngeal reflux”; “extra-esophageal reflux”; “reflux laryngitis”; “COVID-19”; “coronavirus disease 2019”; “COPD”; “chronic obstructive lung disease”; “chronic obstructive pulmonary disease”; “emphysema”; “chronic bronchitis”, and “asthma”* (Supplementary Table 1).

#### Inclusion Criteria

Studies were included if they reported the diagnostic accuracy of at least one liquid biomarker by providing measures of sensitivity, specificity, positive predictive value (PPV), negative predictive value (NPV), and area under the ROC curve (AUC). Studies were required to include human adult subjects with a diagnosis of COPD, asthma, laryngopharyngeal reflux, or COVID-19 infection. Only articles written in English were included due to limited resources for the translation of studies.

#### Exclusion Criteria

Studies that did not report a complete set of diagnostic accuracy measures (i.e., sensitivity, specificity, PPV, NPV, and AUC) for at least one liquid biomarker were excluded. Studies conducted in animal or pediatric populations were excluded. Articles written in languages other than English were also excluded.

#### Screening and Eligibility Assessment of Articles

Sources were assessed in a two steps-process to determine their relevance to this study’s objectives. The first step (screening) included a review of titles and abstracts to discard sources that did not match the inclusion criteria. The second step (eligibility assessment) consisted of a full-text assessment of all remaining articles against the eligibility criteria.

## Results and Discussion

### Source Selection

**FIGURE 3:**
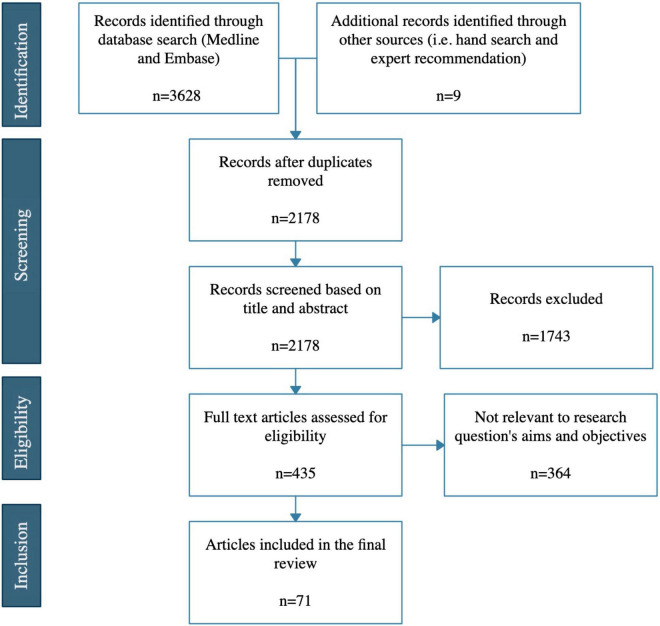
Prisma flow diagram.

The literature search yielded 3,628 results that were imported into the Covidence systematic review software Version v2625 (Veritas Health Innovation, Melbourne, Australia) for screening and eligibility assessment (Figure 3). Nine references were identified by hand-search or by expert recommendations. A total of 1,459 duplicates were removed by the automated software. After the removal of duplicates, 2,178 different studies were screened. The first step of the screening process yielded 435 studies that were assessed for eligibility by full-text reading. After full-text eligibility assessment, 71 studies were included for this review.

### Chronic Obstructive Pulmonary Disease

Chronic obstructive pulmonary disease (COPD) refers to a group of poorly reversible and progressive respiratory conditions, mostly seen in patients with dyspnea, chronic cough, sputum production, and a history of exposure to risk factors of the disease (such as primary or secondary cigarette smoke exposure) (19, 20). The pathophysiology of COPD is associated with multiple factors such as genetic susceptibility, abnormal lung development, cellular senescence, and chronic environmental insults to the lungs, all of which can contribute to the development of chronic pulmonary inflammation (19). Clinical diagnosis of COPD is based on documented persistent airflow limitation on spirometry (19). Inflammatory biomarkers such as high sensitivity C-reactive protein (hs-CRP) have been studied for their ability to diagnose COPD, with moderate specificities (75–83%) but low sensitivities (42–50%) (21) (Table 1).

**TABLE 1:** Diagnostic accuracies of liquid biomarkers for chronic obstructive pulmonary disease (COPD).

Study and country	No of cases	Type of cases	No of controls	Type of controls	Type of biomarker	Biomarker	AUC^a^	Cut-off	Test accuracy indices (%)			
									Sens.^b^	Spec.^c^	PPV^d^	NPV^e^
**Blood**												
Agapakis et al. (33) Greece	81	AECOPD patients	81	Same patients in the stable phase of the disease	Diagnostic biomarker of AECOPD	MPV^f^	0.818	8.2 fL	80	76	78	76
Akiki et al. (45) Lebanon	90	COPD patients	180	Healthy controls	Diagnostic biomarker of COPD	Score based on seven variables	0.890	15.5	76.4	89.3	81	74
	68	Ever smoker COPD patients	180	Healthy controls			0.895	18.5	77.8	88.5	70	82
Andrijevic et al. (34) Serbia	47	AECOPD^g^ patients with left ventricular systolic failure	162	AECOPD patients without left ventricular systolic failure	Diagnostic biomarker of AECOPD complications	NT-proBNP	0.809	1,505 pg/ml	76.6	83.33	57.14	92.47
Antus et al. (25) Hungary	13	Eosinophilic COPD patients	40	Non-eosinophilic COPD patients	Diagnostic biomarker of phenotype	Eosinophils	0.82	0.20 × 10^9^/L	77	76	53	91
							0.78	2.5%	77	63	42	89
Ergan et al. (40) Turkey	15	Bacterial infection in patients with severe AECOPD requiring mechanical ventilation	48	Bacterial infection in patients with severe AECOPD not requiring mechanical ventilation	Prognostic biomarker	PCT^h^ on day 0 (D0)	0.65	0.25 ng/ml	0.65	63	67	45
						PCT on D0 and D3	0.73	0.25 ng/ml	0.73	87	59	52
Li et al. (27) China	48	ACOS^i^ patients	134	COPD patients	Diagnostic biomarker of phenotype	Eosinophils	0.640	0.34 × 10^9^/L	39.6	90.3	59.4	80.7
	24	Steroid-naïve ACOS patients	69	Steroid-naïve COPD patients			0.693	0.29 × 10^9^/L	41.7	94.5	71.4	83.1
	24	ACOS patients ever ICS^j^ users	61	COPD patients ever ICS users			0.588	0.34 × 10^9^/L	41.7	80.3	45.5	77.8
	28	ACOS patients never smokers	55	COPD patients never smokers			0.617	0.29 × 10^9^/L	35.7	92.7	16.4	81.8
	20	ACOS patients ever smokers	79	COPD patients ever smokers			0.692	0.36 × 10^9^/L	45.0	73.4	52.9	86.6
Milkowska-Dymanowska et al. (28) Poland	5	Frequent exacerbator phenotype	14	Non-frequent exacerbator phenotype	Diagnostic biomarker of phenotype	sRAGE^k^	0.81	851 pg/ml	80	93	80	93
Mouronte-Robias et al. (37) Spain	109	COPD patients with lung cancer	83	COPD patients with no lung cancer	Diagnostic biomarker of AECOPD complications	Score based on 3 variables	0.78	3.5 points	80	65.1	43.5	90.7
Sato et al. (41) Japan	27	AECOPD patients with 30-day mortality	168	AECOPD patients with 30-day survival	Prognostic biomarker	MCHC^l^	0.688	31.6 g/dl	59.3	81.0	33.3	92.5
Takayama et al. (26) Japan	56	ACOS patients	65	COPD patients	Diagnostic biomarker of phenotype	Eosinophils	0.637	250/μL	50	82.3	68.6	68.0
	33	Steroid-naïve ACOS patients	57	Steroid-naïve COPD patients			0.677	250/μL	51.4	81.5	65.5	71.0
Taylan et al. (32) Turkey	100	AECOPD patients	100	Same patients in a stable phase of the disease	Diagnostic biomarker of AECOPD	NLR	0.894	3.29	80.8	77.7	72.1	85.1
						CRP	0.814	1.17 mg/dl	71.4	82.3	72.6	81.4
						ESR	0.670	20.5 mm/h	49.2	86.2	69.4	72.9
						Leucocytes	0.771	7,880/μL	71.7	71.2	63.9	77.9
Tilemann et al. (21) Germany	36	COPD patients	174	Healthy controls and non-COPD obstruction	Diagnostic biomarker of COPD	Hs-CRP	0.651	2.39 mg/L	50	75	30	88
								3.5 mg/L	42	83	33	87
Titova et al. (36) Norway	38	AECOPD patients with pulmonary infiltrate	80	AECOPD patients without pulmonary infiltrate	Diagnostic biomarker AECOPD complications	PCT	0.67	0.08 μg/L	63	68	49	79
						CRP	0.73	37 mg/L	66	65	46	81
								45 mg/L	66	71	50	82
								40 mg/L	66	68	48	82
						Leucocytes	0.67	11.0 × 10^9^/L	60	60	40	77
Wang et al. (38) China	90	Non-invasive mechanical ventilation (NIMV) failure in AECOPD patients	286	NIMV success in AECOPD patients	Prognostic biomarker	PCT	0.854	0.31 ng/ml	83.3	83.7	61.61	94.10
						CRP	0.849	15 mg/ml	75.6	93.0	77.23	92.39
Yamaji et al. (42) Japan	31	COPD patients non-responsive to ICS	12	COPD patients responsive to ICS	Prognostic biomarker	Eosinophils	0.65	100/μL	100.0	25.8	34.3	100.0
								200/μL	83.3	37.0	37.0	87.5
								300/μL	50.0	33.3	33.3	76.0
								400/μL	25.0	33.3	33.3	73.5
Yao et al. (39) China	37	AECOPD patients with in-hospital mortality	266	AECOPD patients who survive	Prognostic biomarker	NLR	0.803	6.24	81.08	69.17	26.78	96.34
						PLR	0.639	182.68	64.86	58.27	17.78	92.27
						CRP	0.703	16.45 mg/L	78.39	52.25	18.59	94.56
						NLR +PLR	0.800	–	91.89	60.15	24.29	98.16
						NLR +CRP	0.785	–	89.19	53.01	20.89	97.24
						PLR +CRP	0.694	–	70.27	60.52	19.85	93.60
						NLR +PLR +CRP	0.783	–	89.19	56.77	22.29	97.42
Yilmaz et al. (35) Turkey	19	AECOPD patients with right ventricular failure	40	AECOPD patients without right ventricular failure	Diagnostic biomarker of AECOPD complications	CA-125	0.902	35 U/ml	89.5	85.7	85	90
Zuo et al. (16) China	101	AECOPD patients with pulmonary hypertension	84	AECOPD patients without pulmonary hypertension	Diagnostic biomarker AECOPD complications	NLR	0.701	4.659	81.2	59.5	70.7	72.5
						Platelet: lymphocyte ratio (PLR)	0.669	160.0	77.2	53.6	66.7	66.2
						NT-proBNP	0.776	384 pg/ml	58.4	92.9	90.8	65.0

*^a^AUC: Area under the ROC curve.*

*^b^Sens.: Sensitivity.*

*^c^Spec: Specificity.*

*^d^PPV: Positive predictive value.*

*^e^NPV: Negative predictive value.*

*^f^MPV: Mean platelet volume.*

*^g^AECOPD: Acute exacerbation of COPD.*

*^h^PCT: Procalcitonin.*

*^i^ACOS: Asthma-COPD overlap syndrome.*

*^j^ICS: Inhaled corticosteroids.*

*^k^sRAGE: soluble receptor for advanced glycation end products.*

*^l^MCHC: Mean corpuscular hemoglobin concentration.*

Various COPD classification systems have been proposed over the years, moving away from the classical binary classification of COPD as emphysema and chronic bronchitis (22). Some of these phenotypes include the asthma-COPD overlap syndrome (ACOS), the non-exacerbator phenotype, the frequent exacerbator with emphysema phenotype, and the frequent exacerbator with chronic bronchitis phenotype (22–24). Blood eosinophils can be used to diagnose ACOS and eosinophilic COPD, with sensitivities of 29–55% and 77%, respectively, and specificities of 71–95% and 63–76%, respectively (25–27). Blood sRAGE has been studied for its ability to distinguish the frequent exacerbator COPD phenotype from non-frequent exacerbator COPD phenotype and was found to have a moderate sensitivity of 80%, with a high specificity of 93% (28).

The natural history of COPD is punctuated by periods of acute worsening of respiratory symptoms called acute exacerbations (AECOPD), and a gradual decline in lung function, all of which can eventually lead to death (29). In the US and Canada, the average cost of a severe COPD exacerbation was estimated to be over $18,000 and $9,500, respectively (30, 31). For this reason, there has been a growing interest in identifying biomarkers allowing for early diagnosis of COPD exacerbations. Blood biomarkers, such as neutrophil to lymphocyte ratio (NLR), C-reactive protein (CRP), leukocyte counts, and mean platelet volume, have all been identified as promising biomarkers of COPD exacerbations, with moderate sensitivities (71–80%) and specificities (71–82%) (32, 33).

Other blood biomarkers have been studied for their ability to diagnose complications among AECOPD patients, such as left and right ventricular failure and pulmonary hypertension (34–37). CA-125 was found to be highly accurate for the diagnosis of right ventricular failure, while NT-pro BNP was found to be moderately accurate for the diagnosis of left ventricular systolic failure, with sensitivities of 90 and 70%, and specificities of 86 and 83%, respectively (34, 35). Several other peripheral biomarkers such as procalcitonin, CRP, mean corpuscular hemoglobin concentration (MCHC), and blood cell count ratios have been identified as potential prognostic biomarkers for AECOPD patients (38–41). CRP, neutrophil to lymphocyte ratio (NLR), platelet to lymphocyte ratio (PLR), and MCHC can all be used to predict in-hospital mortality among AECOPD patients with sensitivities ranging from 59 to 89% and specificities ranging from 52 to 81% (38, 39, 41). Procalcitonin can be used to predict non-invasive mechanical ventilation (NIMV) failure in AECOPD patients with a sensitivity of 83% and a specificity of 84% (38). More multicentric research is needed in order to confirm the clinical utility of these biomarkers.

The measurement of COPD exacerbation biomarkers is not yet employed in routine clinical practice. Aside from lung function parameters, very few biomarkers are routinely used for the clinical management of COPD (19). The 2021 GOLD guidelines recommend the utilization of blood eosinophil counts to guide the prescription of inhaled corticosteroids (ICS) for COPD patients (19). Blood eosinophils are among the biomarkers with the best-established diagnostic accuracies in COPD: A blood eosinophil count <100 cells/μL can help to identify patients who are unlikely to respond to treatment with ICS, with a sensitivity of 100%, while a blood eosinophil count >300 cells/μL can help to identify patients with the greatest likelihood of benefiting from treatment with ICS (19, 42).

To date, the implementation of biomarkers in clinical practice for COPD has proven to be difficult, with available data on COPD biomarkers being complex to interpret, largely as a result of weak associations and lack of reproducibility between large patient cohorts (19). Studies varied greatly in terms of sample size, ranging from 5 to 101 COPD cases, biomarker studied (14 different biomarkers or scores evaluated across 18 studies), and biomarker utility (*n* = 2 studies assessing diagnostic biomarkers for COPD, *n* = 2 studies for AECOPD, *n* = 5 studies for AECOPD complications, *n* = 4 studies for COPD phenotype, and *n* = 5 studies for COPD prognostics). The collection of a plethora of detailed genetic and biochemical data from large cohorts (such as COPDGene and SPIROMICS cohorts) have led to an explosion of smaller studies investigating newer biomarkers (e.g., CC16, SP-D, IL-6, fibrinogen, etc.), which unfortunately often remain with uncertain diagnostic accuracies (43–45). Fibrinogen and IL-6, in particular, have been the subject of increasing research interest. However, no studies provided a complete set of diagnostic accuracy measures (i.e., sensitivity, specificity, PPV, NPV, and AUC) at the time of this review. Rigorous and exhaustive diagnostic accuracy studies are warranted to confirm the clinical utility of these biomarkers. In addition, the COPD Biomarker Qualification Consortium has identified several other promising biomarkers, such as desmosine and sRAGE, which can be prioritized for future COPD biomarker research (46). It is hoped that the discovery and implementation of appropriate COPD biomarkers will lead to improved diagnosis, risk stratification, management, and prognosis, thus enabling personalized medicine and leading to better outcomes in COPD patients.

### Asthma

Asthma is one of the most prevalent chronic diseases worldwide (47). Diagnosis is conventionally established based on suggestive clinical symptoms and the evidence of variable expiratory airflow limitation on pulmonary function testing (47).

Although the use of biomarkers for the diagnosis of asthma is rather limited in clinical practice, related studies have been flourishing over the past few years. For instance, atopy-related biomarkers, such as eosinophils, periostin, IgE, and thymus, and activation-regulated chemokine (TARC), and others were proposed for the diagnosis of asthma (12–14, 48–50) (Table 2). For the diagnosis of occupational asthma (OA), sputum eosinophilia following specific inhalation challenges demonstrated high specificities (86–97%) but low sensitivities (57–67%) (51, 52).

**TABLE 2:** Diagnostic accuracies of liquid biomarkers for asthma.

Study and country	No of cases	Type of cases	No of controls	Type of controls	Type of biomarker	Biomarker	AUC^a^	Cut-off	Test accuracy indices (%)			
									Sens.^b^	Spec.^c^	PPV^d^	NPV^e^
**Sputum**												
Berthon et al. (70) Australia	29	Asthma patients unresponsive to oral steroids	25	Asthma patients responsive to oral steroids	Predictive biomarker	Eosinophils	0.776	2.5%	79.2	69.2	70.4	78.3
								4.8%	66.7	76.9	71.4	69.0
						Six-gene signature	0.905	0.36	86.7	76.2	70.6	84.2
								0.63	73.3	95.2	90.1	80.0
Fortuna et al. (13) Spain	22	Asthma patients	28	Non-asthmatic patients	Diagnostic biomarker of non-OA^f^	Eosinophils	0.58	3%	41	75	56	61
Racine et al. (51) Canada	152	Patients with OA (Before SIC)^g^	229	Patients with non-OA (before SIC)	Diagnostic biomarker of OA	Eosinophils	0.61	3%	31	85	41	79
		Patients with OA (After SIC)		Patients with non-OA (After SIC)		Eosinophils	0.82	↑≥3%	57	90	68	85
Smith et al. (12) New-Zealand	17	Asthma patients	30	Symptomatic non-asthmatic patients	Diagnostic biomarker of non-OA	Eosinophils	0.861	3%	86	88	80	92
Suzuki et al. (61) Japan	19	Eosinophilic asthma patients	23	Non-eosinophilic asthma patients	Diagnostic biomarker of phenotype	Basophils	0.896	0.05%	78.9	87	83.4	83.3
								0.10%	63.2	95.7	92.4	75.9
Tsilogianni et al. (15) Greece	31	Patients with ACT^h^< 20	139	Well-controlled asthma (ACT ≥ 20)			0.65	4%	69	61	88	32
	9	Patients with mild to moderate asthma + ACT < 20	114	Well-controlled asthma (ACT ≥ 20)	Monitoring biomarker	Eosinophils	0.58	3%	63	55	94	11
	22	Patients with severe refractory asthma + ACT < 20	25	Well-controlled asthma (ACT ≥ 20)			0.64	4%	68	68	70	65
Vandenplas et al. (52) Belgium	6	Patients with a first – SIC and a second + SIC	29	Patients with a first and second – SIC	Diagnostic biomarker of OA	Eosinophils	0.81	↑ > 3%	67	97	80	93
						Neutrophils	0.74	↑>4%	83	62	31.3	94.7
**Blood**												
Ahmad Al Obaidi et al. (14) Iraq	562	Asthma patients	132	Healthy controls	Diagnostic biomarker of non-OA	IgE	0.96	200 U/ml	93	91	97	86
Berthon et al. (70) Australia	29	Asthma patients unresponsive to oral steroids	25	Asthma patients responsive to oral steroids	Predictive biomarker	Eosinophils	0.775	0.3%	70.8	66.7	68.0	69.6
								0.4%	50	91.7	85.7	64.7
Chang et al. (67) Korea	117	AERD^i^ patients	685	Aspirin-tolerant asthma	Diagnostic biomarker of phenotype	Score based on 14 SNPs^j^	0.821	1.01328	64.7	85	42.1	93.4
Coumou et al. (63) Netherlands	7	Eosinophilic asthma patients	39	Non-eosinophilic asthma patients	Diagnostic biomarker of phenotype	Eosinophils	0.89	0.46 × 10^9^/L	57	97	80	93
Hilvering et al. (66) Netherlands	11	Eosinophilic asthma patients	23	Non-eosinophilic asthma patients	Diagnostic biomarker of phenotype	Model based on 12 variables	0.73	–	76.9	71.4	62.5	83.3
Jia et al. (65) United States	35	Eosinophilic asthma patients	13	Non-eosinophilic asthma patients	Diagnostic biomarker of phenotype	Periostin	0.84	–	57	85	93	37
Kim et al. (50) Korea	61	Patients with isocyanate-induced OA	180	Asymptomatic exposed controls	Diagnostic biomarker of OA	Vit. D binding protein	0.765	311 μg/ml	69	81	55	88
Liang et al. (59) China	124	Eosinophilic asthma patients	68	Non-eosinophilic asthma patients	Diagnostic biomarker of phenotype	Eosinophils	0.698	0.21 × 10^9^/L	67.7	66.2	78.5	52.9
	93	Steroid-naïve eosinophilic asthma patients	46	Steroid-naïve non-eosinophilic asthma patients			0.730	0.19 × 10^9^/L	76.3	67.4	82.6	58.5
Liu et al. (60) China	62	Eosinophilic asthma patients	64	Non-eosinophilic asthma patients	Diagnostic biomarker of phenotype	T2-ILC^k^	0.88	0.076%	67.7	95.3	93.3	75.
						Eosinophils	0.84	55 IU/ml	56.5	70.3	64.8	62.5
						IgE	0.60	0.33 × 10^9^/L	71	85.9	83	75.3
Racine et al. (51) Canada	152	Patients with OA (before SIC)	229	Patients with non-OA (before SIC)	Diagnostic biomarker of OA	Eosinophils	0.61	0.3 × 10^9^/ml	35	79	36	79
Shabana et al. (69) Egypt	N/a	Patients responsive to vitamin D	N/a	Patients non-responsive to vitamin D	Predictive biomarker	IL-17A: IL-10 ratio	0.806	2.66	72.2	83.3	81.25	76.92
Shin et al. (68) Korea	165	AERD patients	397	Aspirin-tolerant asthma patients	Diagnostic biomarker of phenotype	Seven SNPs	0.75	–	34	93	68.2	77.1
Soma et al. (57) Japan	13	Eosinophilic asthma patients	23	Non-eosinophilic asthma patients	Diagnostic biomarker of phenotype	Eosinophils	0.82	0.27 × 10^9^/L	80.0	68.7	76.2	73.3
								0.30 × 10^9^/L	75.0	68.8	75.0	68.8
Suzuki et al. (61) Japan	19	Eosinophilic asthma patients	23	Non-eosinophilic asthma patients	Diagnostic biomarker of phenotype	Eosinophils	0.765	0.25 × 10^9^/L	78.9	69.2	68.2	80
								0.35 × 10^9^/L	47.4	65.	52.9	60
								0.45 × 10^9^/L	26.3	91.3	71.4	60
Tilemann et al. (21) Germany	86	Asthma patients	124	Healthy controls and non-asthmatic obstruction	Diagnostic biomarker of non-OA	Eosinophils	0.602	4.15%	36	83	59	65
						IgE	0.584	90 U/ml	47	73	54	66
Tsilogianni et al. (15) Greece	31	Patients with ACT < 20	139	Well-controlled asthma (ACT ≥ 20)			0.92	156 pg/ml	94	81	95	76
	9	Patients with mild to moderate asthma + ACT < 20	114	Well-controlled asthma (ACT ≥ 20)	Monitoring biomarker	IL-13	0.80	117 pg/ml	88	67	97	32
	22	Patients with severe refractory asthma + ACT < 20	25	Well-controlled asthma (ACT ≥ 20)			0.98	156 pg/ml	92	95	95	91
Vandenplas et al. (49) Belgium	82	Patients with latex-induced OA	25	Symptomatic patients with non-latex induced OA	Diagnostic biomarker of OA	IgE against: NRL extract (K82)	0.84	0.35 kUA/L	94	48	86	71
								1.12 kUA/L	85	76	92	61
								5.41 kUA/L	49	92	95	35
						rHev b 5	0.79	0.35 kUA/L	63	88	94	42
								0.51 kUA/L	62	92	96	43
						rHev b 6.01	0.81	0.35 kUA/L	78	68	89	49
								0.86 kUA/L	68	88	95	46
						rHev b 6.02	0.82	0.35 kUA/L	78	76	91	51
								0.31 kUA/L	79	76	92	53
						rHev b 11	0.72	0.35 kUA/L	34	96	95	28
								0.08 kUA/L	43	92	95	33
Wagener et al. (58) Netherlands	30	Eosinophilic mild to moderate asthma patients	80	Non-eosinophilic mild to moderate asthma patients	Diagnostic biomarker of phenotype	Eosinophils	0.89	0.22 × 10^9^/L	86	79	60	93
						Periostin	0.55	0.25 × 10^9^/L	79	84	64	91
								0.27 × 10^9^/L	78	91	79	91
								26 ng/ml	54	57	29	77
Westerhof et al. (64) Netherlands	116	Eosinophilic asthma patients	220	Non-eosinophilic asthma patients	Diagnostic biomarker of phenotype	Eosinophils	0.83	0.09 × 10^9^/L	96	26	40	92
								0.41 × 10^9^/L	36	95	79	74
						Total IgE	0.69	13.5 kU/L	96	28	41	92
								763.5 kU/L	8	95	47	66
Yormaz et al. (48) Turkey	87	Asthma patients	42	Healthy controls	Diagnostic biomarker of non-OA	TARC^l^	0.934	713.7 ng/L	94.25	85.71	93.2	80
						Periostin	0.792	31.0 ng/ml	91.95	52.38	87.8	75.9
Zhang et al. (62) Australia						Eosinophils	0.898	0.26 × 10^9^/L	83	83	81	85
	71	Eosinophilic asthma patients	67	Non-eosinophilic asthma patients		Eosinophil: lymphocyte ratio (ELR)	0.907	2.7%	92	76	76	92
	25				Diagnostic biomarker of phenotype	Eosinophil: neutrophil ratio (ENR)	0.892	0.10	89.6	74.4	75.8	88.9
		Neutrophilic asthma patients					0.891	0.05	89.6	77.0	77.5	89.3
			113	Non-neutrophilic asthma patients		Eosinophil: macrophage ratio (EMR)	0.898	0.26	98.7	49.4	63.3	97.7
						Neutrophils	0.623	61.5 × 10^9^/L	61.5	63.2	38.1	81.7
						Lymphocytes	0.385	2.54 × 10^9^/L	65.9	48.0	23.8	85.2
						Neutrophil: lymphocyte ratio (NLR)	0.612	1.74	76.9	41.6	29.1	85.3

*^a^AUC: Area under the ROC curve.*

*^b^Sens.: Sensitivity.*

*^c^Spec: Specificity.*

*^d^PPV: Positive predictive value.*

*^e^NPV: Negative predictive value.*

*^f^OA: Occupational asthma.*

*^g^SIC: Specific inhalation challenge.*

*^h^ACT: Asthma control test.*

*^i^ Aspirin-exacerbated respiratory disease.*

*^j^SNP: Single nucleotide polymorphism.*

*^k^T2 ILC: Type 2 Innate Lymphoid cell.*

*^l^TARC: thymus and activation regulated chemokine.*

Asthma is now recognized as a heterogeneous disease characterized by distinctive endotypes and phenotypes. Asthma endotypes can be broadly categorized into T2-high and non-T2 asthma (53) Airway eosinophilia is typically associated with T2-high asthma, while airway neutrophilia is more commonly seen in non-T2 asthma (47). Asthma phenotypes often falling under the T2-high category include early-onset atopic asthma, late-onset eosinophilic asthma, and aspirin-exacerbated respiratory disease (AERD) (53). Asthma phenotypes often falling under the non-T2 category have been categorized according to associated clinical characteristics and include smoking-related asthma, obesity-related asthma, and elderly asthma (53). Of note, this asthma classification model is based on frequent associations between endotypes and clinical manifestations (phenotypes) with known exceptions. A better understanding of asthma biomarkers and the mechanism to which they are a testimony in the pathogenesis of the disease could eventually allow for a more precise classification of asthma and subsequent phenotypes.

Asthma biomarkers have been proposed to classify the disease according to endotypes and phenotypes. Being able to make such classification is notably useful in the health care setting due to their inherent therapeutic and prognostic implications and may provide the basis for personalized medicine (54). While peripheral neutrophil counts are not routinely used in clinical practice, the current international Global Initiative for Asthma (GINA) guidelines recommends the use of eosinophils in the diagnosis and management of moderate or severe asthma to:

(1) Confirm refractory type 2 inflammation (blood or sputum eosinophils).
(2) Assess severe asthma phenotypes (blood or sputum eosinophils).
(3) Adjust treatment for adults with persisting symptoms and/or exacerbations despite high dose ICS or ICS-LABA in the presence of sputum eosinophilia (>3%).
(4) Increase ICS dose independently of the level of symptom control in adults with sputum eosinophilia (>3%).
(5) Guide treatment for adults with moderate to severe asthma who are treated or can be referred to centers experienced with sputum induction testing.
(6) Confirm type-2 inflammatory phenotype in patients refractory to high dose ICS-LABA before prescribing type-2 biologic targeted therapy, such as anti-IL5, anti-IL5R, or anti-IL4R (must have blood eosinophilia but cut-off points may vary according to location) (47).

Sputum eosinophils are generally considered to be reliable biomarkers of Th2 airway inflammation in asthma (47). In order to perform sputum induction, the patient is required to inhale a hypertonic solution by nebulization, which helps to produce sputum that can then be expectorated (55). However, sputum induction testing has the disadvantage of not being readily available in primary care (47). Moreover, sputum induction’s success rate is only around 80%, due to difficulties that may arise during the sampling process (56). For instance, certain patients may experience bronchoconstriction due to the inhalation of hypertonic saline, and others may be unable to produce sufficient saliva for sputum analysis (55).

Blood eosinophils were thus proposed as peripheral surrogate biomarkers for sputum eosinophilia. Overall, studies evaluating the accuracy of blood eosinophils to detect sputum eosinophilia have found moderate to high sensitivities and specificities, ranging from 68 to 86% and 66 to 91%, respectively, for cut-off values between 0.19×10^9^/L and 0.33×10^9^/L (57–62). The use of higher cut-off values for blood eosinophils was associated with higher specificities (91–97%) but lower sensitivities (26–57%) (61, 63, 64). Some other diagnostic biomarkers that have been investigated for T2-high asthma include sputum basophil counts, serum periostin, blood IgE, type-2 innate lymphoid cell counts, and blood cell count ratios (58, 60–62, 64–66).

In addition to asthma endotyping, liquid biomarkers are also used for asthma phenotyping. For the diagnosis of AERD, a T2-high asthma phenotype, it was observed that different combinations of single-nucleotide polymorphism (SNPs) found in blood could differentiate AERD patients from aspirin-tolerant asthma patients with relatively low sensitivities of 34–65%, but high specificities of 85–93% (67, 68). Regarding non-T2 asthma phenotypes, peripheral neutrophil counts, and peripheral lymphocyte counts were found to predict airway neutrophilia with a 61.5 and 65.9% sensitivity, respectively, and a 63.2 and 48% specificity, respectively (62). Their combined ratio, i.e., neutrophil to lymphocyte ratio (NLR), presented a slightly better sensitivity (76.9%) but lower specificity (41.6%) for the prediction of airway neutrophilia (62).

Asthma biomarkers can also be used to predict response to pharmacological treatments (69, 70). Sputum and blood eosinophils have demonstrated sensitivities of 50–79% and specificities of 69–92% for the prediction of responsiveness to oral steroid treatment (70). Finally, asthma biomarkers, such as blood Il-13 and sputum eosinophils, were also proposed to monitor disease state, with highly variable sensitivities (63–94%) and specificities (55–95%) (15).

Included studies were highly heterogenous in terms of population studied, with sample sizes ranging from 6 to 562 cases, and studies including patients with various degrees of asthma control and subtypes of disease, e.g., occupational, and non-occupational, eosinophilic, neutrophilic, or AERD asthma phenotypes, etc. Moreover, few studies evaluated the same biomarkers for the exact same purposes. Overall, 23 different biomarkers or biomarker scores were evaluated across 29 studies. While eosinophils were some of the most frequently studied biomarkers (*n* = 17 studies), they were evaluated for a wide range of clinical purposes ranging from diagnosis of occupational asthma, non-occupational asthma, and asthma phenotype to the prediction of responsiveness to oral steroids, and the monitoring of disease control. This heterogeneity likely contributed to the high degree of variability in diagnostic accuracies reported across the literature.

In recent years, considerable progress has been made in our understanding of asthma with respect to its mechanistic pathways (endotypes) and clinical manifestations (phenotypes). However, these advances have predominantly been made in T2-high asthma, which is reflected in our literature search highlighting mainly type 2 inflammatory biomarkers such as eosinophils, IgE, periostin, T2-ILC, and IL-13. New serum biomarkers such as IL-17, chitinase-3-like protein 1, and ceramide to sphingosine-1-phosphate ratio are also proposed as diagnostic biomarkers for T2-low asthma (71). Large cohort studies have also investigated other T2-high biomarkers such as CCL26, eosinophil-derived neurotoxin, and IL-4, which unfortunately still have uncertain diagnostic accuracies (71). More comprehensive research is still needed to ascertain the diagnostic accuracy, and thus, the clinical utility of these novel biomarkers. Biomarkers in other forms, e.g., the fraction of nitric oxide (FeNO) from exhaled breaths, have well-researched diagnostic accuracies for T2-high asthma. However, gas biomarkers are out of the scope of this review.

Nevertheless, current research is increasingly shifting away from the evaluation of single diagnostic biomarkers toward the multidimensional assessment of a combination of biomarkers (72). New diagnostic algorithms and tools composed of multiple biomarkers can be developed to better classify asthma according to endotype and phenotype, which would considerably increase diagnostic accuracies and be possible *via* microfluidic POC platforms with multiplex capability (72).

### Laryngopharyngeal Reflux

Laryngopharyngeal reflux refers to the retrograde flow of stomach contents into the laryngopharynx, where the refluxate meets the upper aerodigestive tract (73). LPR can be conceptualized as a supra-oesophageal manifestation of gastroesophageal reflux disease (GERD). However, LPR has recently been recognized as a distinct clinical entity from GERD, due to the numerous differences in the pathogenesis, clinical presentation, and outcomes between both diseases (73).

Three different constituents of refluxate are thought to mediate the majority of laryngeal symptoms seen in LPR: hydrochloric acid, pepsin, and bile salts (74). While intragastric pH can be as low as 1.5, the pH of the healthy laryngopharynx is neutral, and its epithelium can easily be damaged by gastric acidity (74). In contrast with the resistant epithelium of the oesophageal mucosa, laryngeal and pharyngeal epitheliums are significantly more vulnerable to damage from gastric acidity. Even a short exposure to gastric refluxate could lead to significant laryngopharyngeal damage (74).

The diagnosis of LPR is usually based on clinical symptoms, suggestive laryngoscopy findings, or 24-h pH monitoring, with or without multichannel intraluminal impedance (MII) (75). The 24-h combined hypopharyngeal-oesophageal multichannel intraluminal impedance with dual pH probe (24h-HEMII-pH) is often considered to be the diagnostic gold standard for LPR for its ability to detect both acid and nonacid reflux (74). It is generally preferred over stand-alone 24-h pH monitoring because approximately half of the patients with LPR may suffer from non-acidic reflux (74, 76). Moreover, when used independently, the proximal and distal oesophageal pH monitoring probes can be unreliable, with at best, a 75 and 50% sensitivity, respectively (76). The oesophageal pH monitoring test, with or without oesophageal impedance, is also not readily available in clinical practice, expensive, and not always tolerated by the patients due to its invasive nature (77, 78).

Laryngoscopic examination is more readily available than 24h-HEMII-pH and is considered to be an important screening tool for the diagnosis of LPR (79). However, laryngoscopic examination for the diagnosis of LPR has been associated with poor sensitivity and specificity of 40 and 50%, respectively (80). Indeed, the laryngoscopic signs associated with LPR are often nonspecific and can also be found in patients with other causes of laryngeal irritation such as phonotrauma, smoking, allergy, infection, and even in healthy individuals (75). Laryngoscopic findings of LPR can also be highly subjective with poor inter-rater reliability between examiners (75, 81). Belafsky’s reflux finding score (RFS), a standardized 8-item scoring system for laryngoscopy findings of LPR, was developed in an attempt to overcome this issue (82). Belafsky’s RFS has shown an increased sensitivity of 87.8% for detecting LPR in comparison with laryngoscopic examination, but its specificity was not improved (37.5%) (74, 83). As for the use of questionnaires, studies have shown variable RSI scores with a specificity ranging between 20 and 83% when used independently for the diagnosis of LPR (84, 85).

It has thus been argued that there is currently no ideal procedure for the diagnosis of LPR, with each existent diagnostic method involving its own limitations (74). For this reason, the use of biomarkers to diagnose LPR has gained considerable attention. Salivary pepsin, in particular, has been identified as a promising diagnostic biomarker for LPR.

Pepsin is a proteinase secreted by the chief cells of the gastric fundus and body (74). It is first secreted as an inactive zymogen called pepsinogen, which gets subsequently activated by gastric acidity (78). In patients with LPR, pepsin is often detectable in the saliva, which is an indicator of recent refluxate in the oropharynx or in the oral cavity where salivary samples are collected (83). However, the sensitivity and specificity of salivary pepsin for the diagnosis of LPR is highly variable and depends on which “gold standard” diagnostic methods were used to establish the corresponding diagnostic accuracy.

When 24-h pH monitoring is used as a gold-standard method to establish the diagnosis of LPR, the sensitivity and specificity of salivary pepsin vary between 42–85% and 28–86%, respectively (86). When establishing the diagnosis of LPR based on clinical symptoms only, the detection of salivary pepsin has a sensitivity of 40–48% and a specificity of 95–98% (11) (Table 3). The limited number of studies focusing on salivary pepsin, their variability in control groups (symptomatic patients vs. otolaryngology clinic patients) and reference tests employed may explain the wide range of reported diagnostic accuracies.

**TABLE 3:** Diagnostic accuracies of liquid biomarkers for laryngopharyngeal reflux (LPR).

Study and country	No of cases	Type of cases	No of controls	Type of controls	Gold standard diagnostic method used for comparison	Type of biomarker	Biomarker	AUC^a^	Cut-off	Test accuracy indices (%)			
										Sens.^b^	Spec.^c^	PPV^d^	NPV^e^
**Saliva**													
Barona-Lleo et al. (11) Spain	180	LPR patients	41	Otolaryngology clinic patients with no LPR	Reflux symptom index (RSI) > 13	Diagnostic biomarker	Fasting pepsin	0.688	Positivity of PEP-test	40	97.56	98.63	27.03
							Fasting and postprandial pepsin	0.715		48.05	95	94.87	48.72
Weitzendorfer et al. (86) Austria	41	LPR patients	29	Symptomatic patients with no LPR	Pathological DeMeester score > 14.72 and > 73 reflux events/24 h	Diagnostic biomarker	Pepsin	0.658	16 ng/ml	85.4	27.6	62.5	57.1
									50 ng/ml	78.1	41.4	65.3	57.1
									100 ng/ml	68.3	58.6	70.0	56.7
									160 ng/ml	53.7	69.0	71.0	51.3
									216 ng/ml	41.5	86.2	81.0	51.0

*^a^AUC: Area under the ROC curve*

*^b^Sens.: Sensitivity*

*^c^Spec: Specificity*

*^d^PPV: Positive predictive value*

*^e^NPV: Negative predictive value*

Due to the imperfect sensitivities and specificities of the gold-standard diagnostic methods used for comparison in these studies, the true diagnostic accuracy of salivary pepsin is difficult to determine. At the same time, the wide variability of salivary pepsin diagnostic accuracy values reported in the literature may complicate the interpretation of test results and may justify advocating for its use in combination with other biomarkers or diagnostic methods. Other emergent biomarkers, such as carbonic anhydrase type III, e-cadherins, mucins, and interleukins were also suggested as potential diagnostic biomarkers for LPR (74). However, to our best knowledge, the diagnostic accuracy of these new biomarkers has not yet been established in large patient cohorts. More research on the role of individual biomarkers and their multidimensional assessment in association with clinical signs, symptoms, and pH impedance is needed in order to develop a robust diagnostic algorithm for LPR (87).

### COVID-19

Since the onset of the COVID-19 pandemic in March 2020, nearly 280 million people have contracted the disease and nearly 5.5 million COVID-19-related deaths have been recorded worldwide (as of January 10th, 2022) (88). In an effort to curb the spread of the disease, unprecedented scholarly efforts have been mobilized to investigate the pathophysiology of the viral infection and to develop rapid diagnostic methods for early detection of the disease. The diagnosis of COVID-19 infection is most commonly made on the basis of nucleic acid amplification testing (NAATs), antigen testing, or serology testing (89). To date, over 1,150 COVID-19 diagnostic tests have been commercialized, among which over a thousand have been approved by regulatory agencies in Europe or North America (90). Of these, 23% are serological tests, 45% are NAAT-based methods for RNA detection, and 31% are antigen detection tests (90). Most of these diagnostic tests have high diagnostic accuracies. A 2021 meta-analysis revealed that combined IgG and IgM serological testing yielded a sensitivity and specificity of 84.5 and 91.6%, respectively (89). Sputum PCR testing presented a sensitivity of 97.2%, with the specificities of most PCR tests being 100%, regardless of sample type (89). Interestingly, peripheral non-specific markers, such as CD169Mo ratio, CRP, and neutrophils have also shown high sensitivities (81–91%) for the diagnosis of COVID-19 infection, but highly variable specificities (55–90%) (91–93) (Table 4).

**TABLE 4:** Diagnostic accuracies of liquid biomarkers for COVID-19 infection.

Study and country	No of cases	Type of cases	No of controls	Type of controls	Type of biomarker	Biomarker	AUC^a^	Cut-off	Test accuracy indices (%)			
									Sens.^b^	Spec.^c^	PPV^d^	NPV^e^
**Blood**												
Ahnach et al. (96) Morocco	44	Severe COVID-19 infection	101	Non-severe COVID-19 infection	Prognostic biomarker of severity	CRP	0.872	10 mg/L	86.36	70.3	55.8	92.21
Albarrán-Sánchez et al. (109) Mexico	83	Death from COVID-19 infection	111	Survival from COVID-19 infection	Prognostic biomarker of mortality	NLR^f^	0.728	12	70.27	69.39	63.41	75.56
Bi et al. (97) China	22	Severe COVID-19 infection	91	Non-severe COVID-19 infection	Prognostic biomarker of severity	Fibrinogen to albumin ratio + platelet count	0.754	–	86.3	59.3	33.9	94.74
Booth et al. (113) United States	43	Death from COVID-19 infection	355	Survival from COVID-19 infection	Prognostic biomarker of mortality	Model based on 5 variables	0.93	–	91	91	62.5	98.4
Chen et al. (102) China	30	Severe COVID-19 infection	58	Mild COVID-19 infection	Prognostic biomarker of severity	PT^g^	0.804	13.35 s	50	92.9	80	76.8
						Thrombin time	0.613	19.85 s	39.1	96.4	81.8	70.5
						D-dimer	0.910	821 ng/ml	84.2	88.2	88.9	88.2
Chen et al. (117) China	104	Death from COVID-19 infection	577	Survival from COVID-19 infection	Prognostic biomarker of mortality	cTn1^h^ + NLR	0.914	–	84	77	46	96
Comins-Boo et al. (91) Spain	24	COVID-19 patients	12	Patients with acute bacterial infections	Diagnostic biomarker	CD169Mo ratio	0.92	3.3	91.67	89.83	78.57	96.36
						CRP	0.758	1.0 mg/dl	91.70	68.50	56.41	94.87
						Neutrophils	0.745	66.5%	91.67	56.52	26.83	97.50
						Lymphocytes	0.770	0.001 × 10^9^/L	73.68	83.87	58.33	91.23
Comins-Boo et al. (91) United States	27	COVID-19 patients who develop VTE^i^	88	COVID-19 patients without VTE	Prognostic biomarker of complications	Maximal D-dimer (J1-J7 of hospitalization)	0.72	1,500 ng/ml	95	37.9	34.5	95.7
								2,000 ng/ml	75	53.4	35.7	86.1
								3,000 ng/ml	70	63.8	40	86.1
								5,000 ng/ml	55	69	38	81.6
Demelo-Rodriguez et al. (119) Spain	23	COVID-19 patients who develop DVT^j^	133	COVID-19 patients without DVT	Prognostic biomarker of complications	D-dimer	0.72	1,570 ng/ml	95.7	29.3	19	97.5
De Michieli et al. (114) Italy	52	Death from COVID-19 infection	374	Survival from COVID-19 infection	Prognostic biomarker of mortality	Model comprised of 8 variables	0.942	–	88.8	88.4	54.4	98
Ding et al. (98) China	30	Severe/life-threatening COVID-19 infection	281	Mild/ordinary COVID-19 infection	Prognostic biomarker of severity	hsCRP^k^ Fibrinogen degradation products	0.850	22.41 mg/L	84.00	73.49	28.1	97.4
								46.42 mg/L	52.00	91.72	40.9	93.9
							0.766	0.95 μg/ml	86.21	53.24	18.6	96.9
								2.57 μg/ml	41.38	91.37	37.2	92.7
Dogan et al. (103) Turkey	20	ICU COVID-19 patients	131	Non-ICU COVID-19 patients	Prognostic biomarker of severity	Procalcitonin	0.86	0.109 ng/ml	85	76	41	96
						Glucose	0.84	114 mg/dl	90	74	34	98
						Urea	0.80	45.7 mg/dl	75	87	47	96
						Creatinine	0.77	1.19 mg/dl	60	92	55	94
						LDH	0.83	315 U/L	69	88	45	96
						Calcium	0.79	8.51 mg/dl	70	86	44	95
						Albumin	0.79	4.13 g/dl	82	69	28	97
						Na^+^	0.69	137 mmol/L	70	73	28	94
						Cl-	0.65	97 mmol/L	44	89	36	92
						CRP	0.85	2.19 mg/dl	95	65	30	99
						Ferritin	0.80	648 ng/ml	75	95	60	97
						Leucocytes	0.73	8.51 × 10^3^/μL	55	85	36	93
						Neutrophils	0.78	4.951 × 10^3^/μL	75	76	32	95
						Lymphocytes	0.66	1.69 × 10^3^/μL	85	48	20	96
						NLR	0.80	2.73	90	59	25	98
						MPV^l^	0.65	10.9 fl	55	74	24	92
						D-dimer	0.86	4,233 ng/ml	79	76	33	96
						Fibrinogen	0.73	354 mg/dl	100	43	16	100
Dujardin et al. (121) Netherlands	53	COVID-19 patients who develop VTE	74	COVID-19 patients without VTE	Prognostic biomarker of complications	D-dimer	0.640	2,000 ng/ml	80	29	53	60
								11,000 ng/ml	37	94	84	63
						CRP	0.752	70 mg/dl	87	49	59	81
								245 mg/dl	43	97	93	67
Feld et al. (115) United States	265	Death from COVID-19 infection	677	Survival from COVID-19 infection	Prognostic biomarker of mortality	Ferritin (day 1)	0.638	799 ng/ml	55.7	60.3	35.6	77.6
						Max. ferritin	0.677	862 ng/ml	74.0	36.4	36.4	82.9
Gregoriano et al. (110) Switzerland	17	Death from COVID-19 infection	72	Survival from COVID-19 infection	Prognostic biomarker of mortality	Mid-regional pro-adrenomedullin	0.78	0.75 nmol/L	92.9	33.3	24.5	95.2
								0.87 nmol/L	92.9	55.0	32.5	97.1
								0.93 nmol/L	92.9	60.0	35.1	97.3
								1.5 nmol/L	42.9	86.7	42.9	86.7
								2.5 nmol/L	21.4	98.3	75.0	84.3
Laguna-Goya et al. (106) Spain	36	Death from COVID-19 infection	465	Survival from COVID-19 infection	Prognostic biomarker of mortality	IL-6	0.74	86 pg/ml	52	89	26	96
						CRP	0.80	8.75 mg/dl	97	53	14	99
						Albumin	0.81	3.4 g/dl	74	78	17	97
						ALT	0.69	25 U/L	80	53	16	96
						LDH	0.78	424 U/L	72	71	16	97
						Ferritin	0.74	1,799 ng/ml	70	75	14	97
						D-dimer	0.75	1,386 ng/ml	62	84	23	96
						Platelet	0.63	245 × 10^3^/μL	63	61	11	95
						Monocyte	0.71	0.4 × 10^3^/μL	77	67	11	96
						Neutrophils	0.76	5.1 × 10^3^/μL	86	62	15	98
						Lymphocytes	0.79	0.9 × 10^3^/μL	63	83	13	98
						NLR	0.83	6.5	86	68	17	98
						Model based on 5 variables	0.94	0.07	88	89	38	99
Li et al. (93) China	458	COVID-19 patients	531	Patients with fever and/or respiratory symptoms	Diagnostic biomarker	Leucocytes	0.539	9.5 × 10^9^/L	95.0	12.8	48.4	74.775.9
						Eosinophils	0.717	0.02 × 10^9^/L	74.7	68.7	67.3	82.7
						Hs-CRP	0.707	4 mg/L	86.7	54.8	62.3	92.5
									99.1	9.2	48.5	
						Eosinophils + leucocytes	0.714	0.02 × 10^9^/L + 9.5 × 10^9^/L	70.5	72.3	68.7	74.0
Luo et al. (104) China	91	Severe/critical COVID-19 infection	59	Ordinary COVID-19 infection	Prognostic biomarker of severity	CRP	0.783	41.3 mg/L	65.0	83.7	81.6	68.2
Luo et al. (104) China	84	Death from COVID-19 infection	214	Survival from COVID-19 infection	Prognostic biomarker of mortality	CRP	0.896	4.14	90.5	77.6	61.3	95.4
Luo et al. China (111)	51	Death from COVID-19 infection	688	Survival from COVID-19 infection	Prognostic biomarker of mortality	IL-2R	0.814	1,220 U/ml	41.18	92.15	28.00	95.48
						IL-6	0.901	39.5 pg/ml	68.63	90.41	34.65	97.49
						IL-8	0.808	30 pg/ml	54.90	90.26	29.47	96.43
						TNF-a	0.724	14.4 pg/ml	33.33	90.12	20.00	94.80
						B cells	0.757	63/μL	39.22	90.70	23.81	95.27
						CD4+ T cells	0.906	323/μL	78.43	90.41	37.74	98.26
						CD8+ T cells	0.905	148/μL	72.55	90.99	37.37	97.81
						NK cells	0.888	81/μL	66.67	90.55	34.34	97.34
						Model based on 3 variables	0.956	0.075	90.20	90.26	40.71	99.20
Outh et al. (92) France	57	COVID-19 patients	64	Hospital patients with negative COVID-19 tests	Diagnostic biomarker	CRP	0.759	36 mg/L	80.7	64.5	67.6	78.4
						Eosinophils	0.852	0.010 g/L	86	79.7	79	86.4
						ENR^m^	0.846	3.344	87.7	73.4	74.6	87.0
						Lymphocytes	0.754	1.520 g/L	91.2	56.3	65	87.8
						LNR^n^	0.621	203.98	59.6	64.1	59.6	64.1
Özyilmaz et al. (116) Turkey	9	Death from COVID-19 infection	96	Survival from COVID-19 infection	Prognostic biomarker of mortality	Troponin 1	0.832	7.8 pg/ml	78	86	77	85
Qin et al. (108) China	178	Death from COVID-19 infection	3,120	Survival from COVID-19 infection	Prognostic biomarker of mortality	Hs-cTn 1	0.78	0.490^a^	63.27	91.06	34	97
						CK-MB^o^	0.71	0.491^a^	60.67	71.82	12	97
						NT-pro BNP	0.81	0.189^a^	91.73	58.80	16	99
						CK	0.67	0.448^a^	64.42	64.24	11	96
						Myoglobin	0.83	0.498^a^	75.00	75.62	21	97
						CRP	0.81	6.545^a^	81.30	66.25	18	98
						D-dimer	0.81	1.126^a^	74.68	72.26	19	97
Rasyid et al. (101) Indonesia	45	ICU COVID-19 patients	250	Non-ICU COVID-19 patients	Prognostic biomarker of severity	Ferritin	0.719	1,288.5 ng/ml	70.5	67	78.8	92.7
						NLR	0.776	4.96	79.5	63.5	81.6	91.3
Rasyid et al. (101) Indonesia	31	Death from COVID-19 infection	264	Survival from COVID-19 infection	Prognostic biomarker of mortality	Ferritin	0.703	1,288.5 ng/ml	69	63.7	78.9	95.1
						NLR	0.764	4.96	67.7	68.9	70.5	94.9
Sharifpour et al. (107) United States	67	Death from COVID-19 infection	201	Survival from COVID-19 infection	Prognostic biomarker of mortality	CRP	0.69	Max. value:				
								150 mg/L	0.955	0.259	0.3	0.95
								350 mg/L	0.418	0.826	0.45	0.81
Tan et al. (99) China	6	Severe COVID-19 infection	21	Mild COVID-19 infection	Prognostic biomarker of severity	Leucocytes	0.51	4.61 × 10^9^/L	83	38	63	89
						Neutrophils	0.57	3.15 × 10^9^/L	83	43	29	90
						Lymphocytes	0.40	1.49 × 10^9^/L	33	67	22	78
						NLR	0.61	2.41	83	43	29	9
						CRP	0.87	20.42 mg/L	83	91	71	95
						ESR^p^	0.78	19.50 mm/L	83	81	56	94
Tang et al. (100) China	28	Severe COVID-19 infection	60	Mild COVID-19 infection	Prognostic biomarker of severity	IL-10	0.53	2.40 pg/ml	42.90	66.70	37.55	71.45
						Leucocytes	0.61	7.08 × 10^9^/L	50	81.70	56.04	77.78
						IL-6	0.67	0.64 pg/ml	89.30	40	40.99	88.90
						Procalcitonin	0.68	0.08 ng/ml	46.40	90.70	97.22	19.47
						Neutrophils	0.69	5.38 × 10^9^/L	57.10	86.70	66.71	81.24
						D-dimer	0.75	238.00 ng/ml	82.10	66	58.99	86.09
						SAA^q^	0.78	17.28 mg/L	96.20	61.70	52.12	97.40
						CRP	0.83	12.26 mg/L	92.9	67.80	57.79	95.27
						Superoxide dismutase	0.89	156.00 kU/L	88.30	89.30	94.65	78.08
Voicu et al. (118) France	40	COVID-19 patients who develop VTE	52	COVID-19 patients without VTE	Prognostic biomarker of complications	D-dimer	0.779	3,300 ng/ml	78	69	66	80
								1,730 ng/ml	100	45	58	100
Wang et al. (112) China	24	Death from COVID-19 infection	175	Survival from COVID-19 infection	Prognostic biomarker of mortality	FAD-85 score based on 3 variables	0.871	85	86.4	81.8	39.6	97.6
Yang et al. (105) China	36	Severe/critical COVID-19 infection	68	Moderate COVID-19 infection	Prognostic biomarker of severity	Calcium	0.59	–	20.0	98.5	87.5	70.2
						Phosphorus	0.71		57.1	84.8	66.7	78.9
						Lymphocytes	0.77		83.3	77.9	66.7	89.8
						ALT	0.62		30.6	88.1	57.9	70.2
						AST	0.64		36.1	92.5	62.8	86.4
						LDH	0.76		78.6	77.0	61.1	88.7
						CRP	0.60		94.4	31.3	42.5	91.3

*^a^AUC: Area under the ROC curve.*

*^b^Sens.: Sensitivity.*

*^c^Spec: Specificity.*

*^d^PPV: Positive predictive value.*

*^e^NPV: Negative predictive value.*

*^f^NLR: neutrophil to lymphocyte ratio.*

*^g^PT: prothrombin time.*

*^h^cTn1: cardiac troponin 1.*

*^i^VTE: venous thromboembolism.*

*^j^DVT: Deep vein thrombosis.*

*^k^hsCRP: High sensitivity C-reactive protein.*

*^l^MPV: Mean platelet volume.*

*^m^ENR: Eosinophil to neutrophil ratio.*

*^n^LNR: Lymphocyte to neutrophil ratio.*

*^o^CK-MB: creatine kinase MB.*

*^p^ESR: Erythrocyte sedimentation rate.*

*^q^SA: Serum amyloid alpha.*

While the development of a vast array of diagnostic tests has allowed for increased rapidity and accuracy of diagnosis, significant progress remains to be made when it comes to the risk-stratification of infected patients. COVID-19 is not only a localized respiratory infection, but also a multisystem inflammatory disease involving complex immunological, inflammatory, and coagulative cascades (94). Of patients contracting COVID-19, a significant proportion will require hospitalization, intensive care unit (ICU) admission, and invasive mechanical ventilation (95). A significant proportion of COVID-19 patients will also develop severe and life-threatening complications such as acute kidney injury, coagulopathies, myocardial infarction, stroke, venous thrombosis, and acute respiratory distress syndrome (95). Our ability to predict which patient will develop severe COVID-19 disease remains limited. Several biomarkers of COVID-19 infection have thus been identified for their ability to predict disease progression and mortality.

Various peripheral blood biomarkers such as serum CRP, D-dimers, fibrinogen, procalcitonin, cytokines, electrolytes, blood cell counts, and blood cell count ratios have been studied for their ability to predict disease progression from non-severe COVID-19 to more severe forms of the disease (96–105). D-dimers have demonstrated moderate to high sensitivities of 82–91% and specificities of 66–88% to predict disease progression (100, 102). Similarly, CRP values between 3.69 and 22.41 mg/dl have been shown to predict disease severity with high sensitivities (83–93%), but inconsistent specificities (50–91%) (96, 98–100). Across different studies, CRP’s sensitivity for the prediction of mortality ranged from 42 to 97%, and its specificity ranged from 23 to 83% (104, 106–108).

Other candidate biomarkers for the prediction of mortality include ferritin, inflammatory cytokines (i.e., IL-2R, IL-6, IL-8, IL-10, TNF-a), troponin 1, mid-regional proadrenomedullin, blood cell counts, and blood cell count ratios (101, 102, 106–117). High levels of ferritin (1,288.5–1,799 ng/ml) have demonstrated a moderate diagnostic accuracy for the prediction of mortality with a sensitivity of approximately 70% and a specificity of 64–75% (101, 104). Lower ferritin cut-off values were less promising in terms of sensitivity (56–74%) and specificity (36–60%) (115). Increased serum D-dimers were also able to predict COVID-19 mortality with moderate sensitivities of 62–75% and specificities ranging from 72 to 84%.(106, 108) Additionally, D-dimer levels between 1,500 and 3,300 ng/ml have shown moderate to high sensitivities (70–96%) for the prediction of thrombotic events in COVID-19 patients. However, the associated specificities remained low to moderate, ranging from 22 to 69% (118–121).

To date, most risk-stratification clinical tools for the prediction of COVID-19 severity have used an imperfect combination of few clinical and biological variables. There is an urgent need for the development of accurate risk-stratification algorithms allowing for the early identification of patients at risk of severe COVID-19 disease, particularly in a context of limited human and material resources. More research on prognosis biomarkers of COVID-19 will allow for better integration of clinical and laboratory data and the creation of comprehensive and accurate risk-stratification algorithms. These risk-stratification systems could play a decisive role in the planning of patient management and future utilization of available resources.

### Challenges in Translating Biomarkers From Discovery to Clinical Use

**FIGURE 4:**
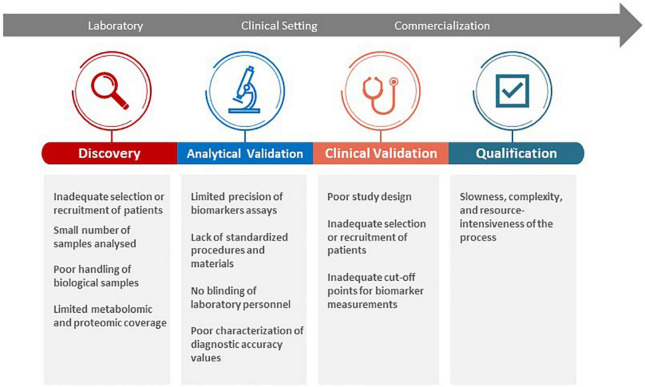
Challenges in biomarker discovery, validation, and qualification for clinical translation.

As the field of liquid biomarkers continues to advance, novel biomarkers are discovered with uncertain diagnostic accuracies or cohort reproducibility (17). In the past, very few guidelines or protocols have been developed to oversee the direction of such research. Scientific data is still considered relatively insufficient to fully support the use of most biomarkers in clinical settings. In light of these challenges, health regulatory bodies (e.g., the U.S. Food and Drug Administration) and funding agencies (e.g., the Foundation for the National Institutes of Health) have developed a Biomarker Qualification Program and a Biomarkers Consortium, respectively, to facilitate biomarker development and standardization for clinical diagnostics and drug monitoring (122, 123). For translation into clinical practice, a biomarker needs to fulfill specific clinical and industrial standards through a process of discovery, analytical validation, clinical validation, and be reviewed by relevant health authorities for qualification (124). A rigorous validation and qualification process is necessary, but also leads to multiple scientific and institutional challenges for clinical translation (Figure 4). Nevertheless, relevant stakeholders (e.g., researchers, clinicians, patients) should be made aware of these limitations and ensure that practical steps (e.g., reporting both positive and negative findings, using robust analytical techniques) are taken in the early development phase of biomarkers (17, 125–127). For instance, analytical validation involves the assessment of the test platform used to measure the biomarker in terms of accuracy, reproducibility, dynamic range, and variability (124). Clinical validation is needed to ensure the consistency and accuracy of the test in predicting the clinical outcome that it is intended to reflect and further characterizes diagnostic accuracy (124). Qualification allows for the identification of biomarkers that can be relied on for a specific interpretation and application within a specific context of use by relevant health authorities (124, 128). As such, a prioritization of biomarkers to be used with defined purposes (e.g., disease phenotyping vs. risk stratification) in specific contexts is recommended at an early stage of the biomarker development process to facilitate validation and qualification.

### Future Prospect of Liquid Biomarkers in Airway Diseases Point-of-Care Applications

Current clinical biomarker detection methods vary based on the health condition tested for and the specific markers of interest. For example, LPR detection commonly utilizes enzyme-linked immunosorbent assays (ELISAs) and lateral flow assays (LFAs) such as the Peptest ^®^ to monitor pH levels and pepsin concentration (129). Similarly, COVID-19 diagnosis relies primarily on various NAATs such as real-time reverse-transcription PCR or loop-mediated isothermal amplification (LAMP), but also leveraging antigen/antibody testing via ELISAs, LFAs, neutralization bioassays, and chemiluminescent immunoassays (130, 131). Each format has associated advantages (e.g., speed, multiplexing, automation) and disadvantages (e.g., trained personnel and dedicated laboratories). For instance, traditional ELISAs take several hours to complete and require dedicated equipment and trained personnel, which limits same-day diagnosis capacity for clinics lacking such resources. In contrast, LFAs offer rapid testing but suffer from reduced sensitivity and accuracy that may necessitate further validation for a reliable diagnosis (132, 133). Additionally, LFAs results are generally qualitative or semi-quantitative, which can be problematic for diseases that require accurate, quantitative results to provide a correct diagnosis and treatment plan (134–136). Irrespective of the test format, the sample requirements (acquisition/preparation/volume) can vary significantly. In some cases, sample acquisition can cause notable patient discomfort such as the mid-turbinate swab required for certain COVID-19 tests (131). The physical properties of a sample may also dictate the speed of the assay with, for example, viscous samples prolonging analysis (137).

To address the limitations of current methods, novel microfluidic detection devices are being actively explored to develop POC testing capable of delivering accurate, rapid, inexpensive diagnoses (138–142). Microfluidic devices include microfabricated fluid channels capable of efficiently manipulating small amounts of fluids for biochemical reactions (143). It should be acknowledged that although LFAs do not fit this conventional definition, they do have a root in microfluidics (144). These microfluidic platforms possess several critical advantages for in-vitro diagnostics over conventional clinical practices (including LFAs). Firstly, they can handle small sample volumes with significant spatial-temporal accuracy in a high-throughput manner—notable criteria of major diagnostic companies (130, 145). The improved sensing parameters those microfluidic devices provide are partly attributable to the precise control of fluid quantities and sample/reagent flow rates, which enable the separation and detection of analytes with high accuracy and sensitivity (146). Furthermore, these systems can deliver diagnostic results within minutes and are generally fabricated from inexpensive polymers (e.g., PDMS or polycarbonate) or cellulose (e.g., paper or thread) substrates (147–150). Finally, microfluidic platforms offer the distinct benefit of having multiplexing capacity. For example, multiple biomarkers of a given disease or infection can be detected simultaneously through several channels of a single device, which could significantly enhance the accuracy of diagnosis and reduce the risk of false-positive and false-negative results (151). An alternative multiplexing method developed combined bead capture and sonic mixing technology into a single device to detect cancer biomarkers within 20 min at a sensitivity level up to 0.028 ng/mL (147). Notably, this device had the capacity to analyze up to 500 biomarkers simultaneously (147, 152). A further multiplex microfluidic detection system used microfluidic cassettes, in combination with microarray technology, to detect tumor markers and demonstrated comparable results with standard, slower clinical instruments (148). As no liquid exchange occurs between the disposable cassette and handheld device, cross-contamination between samples is minimized. Fundamentally, these examples demonstrate the significant scope and flexibility of different microfluidic technologies that can be harnessed to develop multiplexed detection systems.

Microfluidic devices have been proposed to analyze biomarkers present in various biofluids including saliva, sputum, and blood (153–156). Blood has traditionally been the predominantly studied fluid in biomarker research due to the simplicity of sample collection and analysis. Most biomarkers with well-established diagnostic accuracy for COPD, asthma, LPR, and COVID-19 are thus still from blood, rather than from saliva, sputum, or other local surface fluids, with the exception of a few sputum biomarkers in asthma, and saliva biomarkers in LPR. In fact, saliva- and sputum-based biomarkers have been increasingly recognized as “a window on health status” in molecular diagnostics (157). For instance, laryngeal surface secretions have been evaluated for injury-induced inflammation of vocal folds in humans (158). Further efforts to establish the reliability and accuracy of saliva and sputum-based biomarkers of airway diseases will fulfill a true promise of point-of-care diagnosis for patients and clinicians.

By analyzing specific biomarkers, several devices have been applied for diagnosing asthma, COPD, and COVID-19 (156, 159, 160). For instance, a recently developed COVID-19 diagnostic tool utilized a microfluidic chip and Raman spectroscopy to provide an integrated platform that can trap viruses from biological fluids and identify patient infection status within minutes (161). A notable advantage of this system is that, as the detection is based upon the physical properties of the virus, accurate COVID-19 detection is possible across multiple variants. Equally, the platform could be readily adjusted to capture other viruses or bacteria relevant for other infections. Comprehensive reviews of the growing number of microfluidic devices applied for biomarker analysis are available elsewhere with an understanding that very limited products reached commercialization standards (139, 141, 145, 162–165).

### Limitations to Point-of-Care Implementation

The simplicity of POC technology is critical to promoting its accessibility and devolving testing away from laboratories to help streamline the future of diagnostic technology (166). As these devices are intended to be operable by both patients and clinicians alike, it is critical that no convoluted training and equipment is required for their use (167). Research efforts continue to target the development of devices capable of delivering accurate results in a compressed timescale. For instance, the SARS-CoV-2 lateral flow rapid tests that have become widely available enable users to perform a simple self-test at home and achieve a result within 15 min. However, it should be noted that whilst huge advances have been made in rapid, self-testing since the onset of the SARS-CoV-2 pandemic, RT-PCR analysis remains the gold standard for diagnosis. In the case of SARS-CoV-2 testing, one study found that four different kits reported sensitivity values only in the range of 44.6–54.9% (168). In comparison, RT-PCR has previously been found to have a sensitivity of 97.2% when testing sputum samples (89). As such, the sensitivity of rapid testing currently limits it to functioning as an initial diagnosis that requires further confirmation via conventional laboratory testing.

Point-of-care research also aims to diversify the output that a single sample can provide by expanding the multiplex capacity of testing platforms (169). If multiplex devices with high fidelity become widely available, POC technology can help minimize diagnostic wait times and enable patients to receive treatment far sooner than sequential, lengthy testing procedures would otherwise allow (170). The access of multiplex POC technology will be a gamechanger for chronic and complex disease diagnosis and monitoring. Unfortunately, a drawback of multiplex devices is that they are much more susceptible to cross-contamination, which could severely impair the accuracy of the result (171). To address this, device architecture can be modified with, for example, hydrophobic barriers that ensure reagents and analytes are restricted to flow along a defined path only that prevents mixing (172, 173).

Aside from the technical challenges that remain for POC testing, administrative obstacles must be realized and overcome to deploy POC devices to the general public. A top-down approach driven by a limited number of health professionals has proven ineffective in practice (174). Establishing an effective POC testing network requires a well-structured, multi-disciplinary governance encompassing both clinical and managerial support. For instance, allocating human resources of staff training on POC devices is needed to keep the POC testing network sustainable. Communicating the availability of POC testing to patients is also critical in ensuring a high uptake of community use. This is notably true for promoting its use in isolated rural areas where the introduction of POC testing is predicted to have the greatest impact (175).

## Conclusion

The landscape of biomarkers for airway diseases remains extremely vast and highly heterogeneous. Nonetheless, research efforts and financial investments have facilitated the discovery of a variety of promising biomarkers, among which a few have well-circumscribed diagnostic accuracies and can reliably be used clinically. For communicable airway diseases like COVID-19, diagnosis is largely based on direct detection of the viral load whilst supplementary biomarkers are needed for risk stratification and severity prediction. For non-communicable airway diseases, liquid biomarkers have various clinical applications. For instance, sputum and blood eosinophils can be used for the phenotyping and endotyping of asthma, blood eosinophils to guide management of COPD, and salivary pepsin to diagnose LPR.

Despite considerable progress, the majority of microfluidic POC devices remain at a pre-clinical, developmental stage. The lack of standardization across device fabrication and the difficulty in incorporating multiple testing components into a single device present obstacle for manufacturing and scaling-up production. From a testing standpoint, the technical complexity and validation challenges remain limiting factors for device translation to clinical settings. That said, the field of diagnostics continues to transition toward detection devices based on ease of use, low sample volume, and rapid results. Given the ongoing drive to develop microfluidic systems that are simple to use and sensitive, microfluidic systems will continue to trend toward becoming an indispensable part of the healthcare industry as a critical element of POC-based disease diagnosis and pathogen detection in airway diseases.

## Author Contributions

VL and NL-J conceived the manuscript. VL, PC, and NL-J wrote the manuscript. VL designed the figures and tables. All authors read and approved the manuscript.

## Author Disclaimer

The presented content is solely the responsibility of the authors and does not necessarily represent the official views of the above funding agencies.

## Conflict of Interest

The authors declare that the research was conducted in the absence of any commercial or financial relationships that could be construed as a potential conflict of interest.

## Publisher’s Note

All claims expressed in this article are solely those of the authors and do not necessarily represent those of their affiliated organizations, or those of the publisher, the editors and the reviewers. Any product that may be evaluated in this article, or claim that may be made by its manufacturer, is not guaranteed or endorsed by the publisher.
