# Supplementary material for: Diagnostic Accuracy of Liquid Biomarkers in Airway Diseases: Toward Point-of-Care Applications
Source: Front Med (Lausanne). 2022 Jun 6;9:855250. doi: 10.3389/fmed.2022.855250 (PMC9207186; doi:10.3389/fmed.2022.855250)
Supplement: Supplementary file 1 [file Table_1.docx]

**Table S1. Search Strategy for Embase and Medline Databases.**

| **Database**: Embase | **Dates:** June 22^nd^ 2021 |
| --- | --- |
| **Keywords related to populations** | laryngopharyngeal reflux.mp. OR laryngopharyngeal reflux/ OR proximal reflux.mp. OR hypopharyngeal reflux.mp. OR extra-esophageal reflux.mp. OR reflux laryngitis.mp.  copd.mp. OR exp chronic obstructive lung disease/ OR chronic obstructive pulmonary disease.mp. OR emphysema.mp. OR emphysema/ OR chronic bronchitis.mp. OR chronic bronchitis/  exp asthma/ OR asthma.mp.  Covid-19.mp. or coronavirus disease 2019/ |
| **AND** | |
| **Keywords related to the outcome measurement** | diagnostic accuracy/ OR "sensitivity and specificity"/ OR sensitivit*.mp.OR specificit*.mp. |
| **AND** | |
| **Keywords related to Biomarkers** | exp biological marker/ OR biomarker*.mp. OR biologic* marker*.mp. |
|  | |
| **Database**: Medline | **Dates:** June 22^nd^ 2021 |
| **Keywords related to populations** | laryngopharyngeal reflux.mp. OR laryngopharyngeal reflux/ OR proximal reflux.mp. OR hypopharyngeal reflux.mp. OR extra-esophageal reflux.mp. OR reflux laryngitis.mp.  copd.mp. OR exp chronic obstructive lung disease/ OR chronic obstructive pulmonary disease.mp. OR emphysema.mp. OR emphysema/ OR chronic bronchitis.mp. OR chronic bronchitis/  exp asthma/ OR asthma.mp.  Covid-19.mp. or coronavirus disease 2019/ |
| **AND** | |
| **Keywords related to the outcome measurement** | exp "sensitivity and specificity"/ OR sensitivit*.mp.OR specificit*.mp.OR diagnostic accuracy.mp. |
| **AND** | |
| **Keywords related to Biomarkers** | exp biological marker/ OR biomarker*.mp. OR biologic* marker*.mp. |
